# Evaluation of Flu Vaccination Coverage among Healthcare Workers during a 3 Years’ Study Period and Attitude towards Influenza and Potential COVID-19 Vaccination in the Context of the Pandemic

**DOI:** 10.3390/vaccines9070769

**Published:** 2021-07-09

**Authors:** Giuditta Scardina, Luca Ceccarelli, Virginia Casigliani, Sara Mazzilli, Marco Napoletano, Martina Padovan, Armando Petillo, Daniele Sironi, Cinzia Brilli, Vittorio Gattini, Lara Tavoschi, Rudy Foddis, Giovanni Guglielmi, Gaetano Pierpaolo Privitera, Angelo Baggiani

**Affiliations:** 1 Department of Translational Research and New Technologies in Medicine and Surgery, University of Pisa, 56121 Pisa, Italy; giudittascardina7@yahoo.it (G.S.); v.casigliani7@gmail.com (V.C.); sara.mazzill@gmail.com (S.M.); marco.napoletano@ao-pisa.toscana.it (M.N.); padovan.martina@gmail.com (M.P.); armando.petillo@virgilio.it (A.P.); daniele.sironi@outlook.it (D.S.); lara.tavoschi@unipi.it (L.T.); rudy.foddis@med.unipi.it (R.F.); gaetano.privitera@unipi.it (G.P.P.); angelo.baggiani@med.unipi.it (A.B.); 2 Scuola Normale Superiore di Pisa, 56121 Pisa, Italy; 3 Division of Occupational & Preventive Medicine, Santa Chiara Hospital, 56121 Pisa, Italy; c.brilli@ao-pisa.toscana.it (C.B.); v.gattini@ao-pisa.toscana.it (V.G.); g.guglielmi@ao-pisa.toscana.it (G.G.)

**Keywords:** healthcare workers, vaccine hesitancy, vaccine coverage, flu vaccination campaigns, COVID-19 vaccine

## Abstract

(1) Background: vaccination of healthcare workers (HCWs) against seasonal influenza is considered the most effective way to protect HCWs, ensure patient’s safety and to maintain essential health care services during influenza epidemics. With the present study we aimed to evaluate the efficacy of incremental bundles of measures implemented during the last three flu campaigns and to assess the attitudes towards influenza vaccination and a potential vaccine against COVID-19 among HCWs, in a large university hospital in Pisa, Italy. (2) Methods: We described measures implemented during 2018/2019, 2019/2020 and 2020/2021 and assessed their impact on flu vaccine coverage (VC) among employees and residents in Pisa university hospital. We considered sex, profession and ward to investigate differences in uptake. In addition, in 2020 a survey was developed and distributed to all employees to evaluate flu and COVID-19 vaccines attitudes. (3) Results: during the 2018/19 and 2019/20 flu campaigns the overall VC rate among HCWs was, respectively, 10.2% and 11.9%. In 2020/21 the overall VC rate jumped to 39.3% (+ 230.6%). Results from the survey indicated a more positive attitude towards flu vaccine as compared to COVID-19 vaccines among the 10.6% of the staff members who responded to the survey. In addition, 70.97% of HCWs totally agreed that being vaccinated against influenza would be more important than the previous years because of COVID-19 emergency. (4) Conclusions: a significant increase in VC was observed in 2020/21, especially among those sub-groups with consistently lower uptake in previous years. The COVID-19 pandemic positively influenced flu vaccination uptake during the 2020/21 season.

## 1. Introduction

Influenza represents a serious public health issue, both in clinical, epidemiological and socio-economic terms. It is responsible for a significant burden of morbidity and mortality, and high direct and indirect costs, deriving from hospitalization and absenteeism at work [1]. 

Compared with the general population, healthcare workers (HCWs) are at higher risk of contracting influenza and of spreading it to other colleagues or patients, who may be especially vulnerable to complications (e.g., infants, the elderly and individuals with underlying conditions or immunosuppressed) [2,3]. Vaccination of HCWs against seasonal influenza is considered the most effective way to protect HCWs, to ensure patient’s safety and to maintain essential healthcare services during influenza epidemics [4,5]. Although annual seasonal flu vaccination (SIV) for all or some HCWs is highly recommended in all the countries of the WHO European region, including Italy, data on SIV coverage in HCWs is not always available and varies widely across countries, remaining far below the recommended 75% target [6]. For instance, in the 2015–16, 2016–17 and 2017–18 seasons, only 12 Countries of the WHO European Region provided flu vaccination coverage (VC) rates among HCWs, which varied from 15.6% (Italy) to 63.2% (Belgium), with a median of 30.2% [7]. In Italy, there is no official data on flu vaccination coverage among HCWs [8]. However, two systematic reviews estimated a flu VC close to 13% for nurses [9] and to 23% for physicians [10]. Such low levels of vaccination coverage among HCWs could be at least partly attributed to the increasing phenomenon of vaccine hesitancy (VH), defined as a delay in acceptance or refusal of vaccines despite availability of vaccination services [11]. In 2011, the “3Cs’’ model was proposed by the WHO EURO Vaccine Communications Working Group, recognizing three main domains of factors that could influence VH: complacency, whether vaccination is necessary or not; convenience, access to vaccination; and confidence, trust in its effectiveness [12]. Multiple studies have highlighted that despite being reported as the main source of vaccine information for the public [13], HCWs can be hesitant themselves [11].

Before the beginning of the 2020–2021 flu vaccination campaign, it was well known that seasonal flu vaccination among HCWs would have been particularly important this year, due to possible co-circulation of influenza viruses and SARS-CoV-2 [14]. HCWs are the professional category most exposed to SARS-CoV-2 infection, working on the front line, often in conditions of shortage of personnel and resources, in overloaded hospitals [15]. Up to 7 April 2021, 129,873 HCWs got infected in Italy, representing 10% of all confirmed cases [16]. Parallel to ongoing efforts to control the spread of the virus, availability and access to COVID-19 vaccines plays a crucial role in protecting healthcare staff and preventing nosocomial outbreaks. However, COVID-19 vaccination attitude among HCWs is still unclear [17].

With the present study we aimed firstly to evaluate the efficacy of the measures implemented during the flu vaccination campaigns of 2018/2019, 2019/2020 and 2020/2021 flu seasons in a teaching hospital in Tuscany (Italy) through the assessment of VC among the HCWs. In addition, we assessed knowledge, beliefs and attitudes towards flu vaccination and a potential vaccine against COVID-19-prior to any vaccine licensure among the HCWs, through the development and distribution of an ad hoc questionnaire.

## 2. Materials and Methods

### 2.1. Setting and Study Population

The Azienda Ospedaliero-Universitaria Pisana (AOUP) is a large teaching tertiary hospital, located in Pisa, Tuscany, which represents the referral hospital for the North-West area of Tuscany, with 1146 beds [18]. It is constituted of 10 wards and 158 buildings, with over 5000 employees and about 1000 residents.

### 2.2. Flu Campaign 2018/19

Since the 2018/19 flu season, targeted initiatives aimed at increasing flu VC among AOUP HCWs were carried out by the Hygiene and Epidemiology and Occupational Medicine units, in close collaboration with the Medical Directorate. Different strategies based on promoting and facilitating access to vaccination have been progressively implemented during the following seasons. During 2018/19 flu vaccination campaign, promotion materials (e.g., posters and flyers) were made available in the common areas of each unit and access to vaccination was facilitated through the set-up of an On-Site Vaccination (OSV) intervention, complementing the two Occupational Medicine vaccination clinics with ad hoc vaccination services in several wards. 

### 2.3. Flu Campaign 2019/20

In addition to such measures, in the 2019/20 campaign, invitations to get vaccinated were emailed to each employee. The Hygiene and Epidemiology and Occupational Medicine units organized meetings with the medical and nursing staff to inform them about benefits and modalities of the flu vaccination campaign and to urge them to promote vaccination among ward staff members. Additionally, an opening ceremony during which the general director and other high-ranking AOUP staff members received immunization, was organized and publicized through the AOUP website and local TV channels and newspapers. 

### 2.4. Flu Campaign 2020/21

Due to the COVID-19 pandemic in July 2020, a task force (TF) was created to plan the 2020/2021 flu vaccination campaign. To better understand factors associated with flu vaccination uptake among HCWs, the TF developed a survey to collect information on attitudes towards SIV and vaccination against COVID-19 to inform the design of the Campaign. Information collected through the survey was reported to the TF in mid-September and presented in the AOUP website. In addition to the measures rolled-out during past seasons, in 2020/21 the number of OSV was expanded, opening hours were extended, professionals delivering vaccination services increased and education and training sessions were organized for AOUP staff (details in Table 1). A reminder letter was sent to all the employees and residents at the start of the flu vaccination campaign on 12 October 2020, providing information about the immunization plan.

### 2.5. Development and Distribution of the Survey

The self-administered anonymous survey consisted of 14 items grouped in four parts, each collecting information about:

1. Socio-demographic characteristics of the participants, professional category, type of ward and having worked in a dedicated COVID-19 area during the first phase of the pandemic;
2. Flu vaccination attitude, risk perception of having the flu compared to the general population, previous season flu vaccine uptake and intention to get the flu jab in 2020/21 season;
3. COVID-19 vaccination attitude and intention to get the vaccine;
4. Perceived higher importance of flu vaccine during the 2020/21 in comparison to previous years in the context of the pandemic (“impact of pandemic on flu vaccine attitude” variable);
5. Suggestions for the 2020/21 campaign.

The survey included both categorical and 5-points Likert scale questions (2 levels of agreement, 1 neutral choice, 2 levels of disagreement). Between August and September 2020, the survey was sent via email using the company/university mailbox to all the employees and residents of the AOUP and was promoted in the hospital website.

In order to evaluate the flu vaccination attitude, we adapted the 12 items MoVac-flu scale developed by Vallée-Tourangeau et al. [19]. The three dimensions of vaccine hesitancy (complacency, convenience, confidence) were studied through questions adapted from the study of Quinn et al. [20]. These items were modified to explore such dimensions for COVID-19 vaccination too. An additional ad hoc item (“impact of pandemic on flu vaccine attitude”) was created to evaluate the impact of the pandemic on the attitude towards the flu vaccine. 

In order to evaluate the attitude toward the flu vaccine (“Flu vaccination attitude”) and the COVID-19 vaccine (“COVID-19 vaccination attitude”), two scores were generated by the sum of Likert-scale items. For both scores, the range varied from 5 to 25 points, with higher scores corresponding to a more positive attitude.

The full questionnaire, translated in English, is available in the Supplementary Materials.

### 2.6. Data Collection and Assessment of Vaccination Coverage

During each flu vaccination season the Occupational Medicine unit collected data on HCWs’ age, gender, professional category and vaccination status. Data was stored in an electronic database (Microsoft Excel) at the end of each flu season (starting from the 2018/19 season). In order to calculate SIV vaccination coverage during the three flu seasons, the total number of AOUP employees, stratified by professional category, and the total number of residents at 31st December of each year were used for denominators. 

### 2.7. Statistical Analysis

#### 2.7.1. Analysis of the Vaccination Coverage Rates during the Three Flu Seasons

At the end of each flu season, comparison between each potential risk factor categories that might have an influence on HCWs vaccine uptake were carried out using the Chi-square test of Pearson or Fisher exact test, in case any expected frequency was lower than five. Then, a univariate analysis was carried out to explore the association between each independent variable and the different outcome of interest (having received flu vaccine) using logistic regression. All independent variables found to be associated at *p*-value less than 0.05 during the univariate analyses were entered in the multivariate logistic regression. Finally, a multivariate logistic regression model was constructed to identify factors significantly and independently associated with the binary outcome variable.

To build multivariate models a manual stepwise variables’ selection procedure was used, in order to assess confounding and effect modification. To select the variables included in the models, we ran the Likelihood-ratio test. All reported values are two-sided, and a value of *p* ≤ 0.05 was used as a threshold for statistical significance for all analyses. In addition, to better investigate changes before and after each campaign, the percentage variations among the three seasons were calculated. 

#### 2.7.2. Analysis of the Survey

First, a descriptive analysis of the main sample characteristics was conducted. Then, we explored the association between each independent variable and the outcomes of interest, “flu vaccination attitude” and “COVID-19 vaccination attitude” in three stages. 

Mann–Whitney test and Kruskal–Wallis test were used to find out the significant associations between each independent variable and the different outcomes of interest.

Then, a univariate analysis was carried out using linear regression. Finally, the variables found to be significantly associated (*p* ≤ 0.05) were entered in the multivariate linear regression model. 

To build multivariate models a manual stepwise variables’ selection procedure was used, in order to assess confounding and effect modification. All reported values are two-sided, and a value of *p* ≤ 0.05 was used as a threshold for statistical significance for all analyses. Data analysis was carried out using the software Stata (version 13.0).

## 3. Results

Starting from the 2018–2019 campaign, an increasing flu VC rate among HCWs was registered. However, while in the 2019–2020 campaign the increase was minimal (∆% = 23.1), in the 2020–2021 campaign a significant increase was observed compared with the previous year (∆% = 177.6).

Characteristics of the population investigated during the 3 following seasons (2018–2019, 2019–2020 and 2020–2021) are shown in Table 2; the results of the multivariate analysis (one for each flu vaccination campaign) are presented in Table 3.

### 3.1. The 2018–2019 Flu Vaccination Campaign

During the 2018–2019 vaccination campaign there were 5721 HCWs working at the AOUP; 3968 (69.4%) were females. At the end of the 2018–2019 season, the vaccination coverage rate against influenza among HCWs was 11.6% (663). According to the multivariate logistic regression model, HCWs aged more than 60 had a higher likelihood (OR: 1.65–95% CI: 1.07–2.55) of having received the immunization when compared with younger HCWs. Females (OR: 0.67–95% CI: 0.57–0.80) and nurses (OR: 0.30–95% CI: 0.24–0.38) had a lower likelihood of being vaccinated when compared with males and physicians, residents and administrative staff. 

### 3.2. The 2019–2020 Flu Vaccination Campaign

During the 2019–2020 vaccination campaign there were 5936 HCWs working at the AOUP; 4064 (68.4%) were females. At the end of the 2019–2020 season, the vaccination coverage rate against influenza among HCWs was 14.3% (847) with a ∆% of plus 23.1 compared to the previous year. Among the 663 HCWs already immunized during the 2018–2019 season, 362 (54.6%) received the vaccination also during the 2019–2020 campaign. According to the multivariate logistic regression model, HCWs aged less than 30 had a higher likelihood of having received the immunization when compared with older HCWs. Nurses (OR: 0.39–95% CI: 0.31–0.51) and other HCWs (OR: 0.41–95% CI: 0.31–0.54) had a lower likelihood of being vaccinated when compared with physicians and residents. People already vaccinated in the previous flu season had a high likelihood of being vaccinated compared with people not immunized in 2018–19 (OR: 12.84–95% CI: 10.60–15.56).

### 3.3. The 2020–2021 Flu Vaccination Campaign

During the 2020–2021 vaccination campaign there were 6323 HCWs working at the AOUP; 4333 (68.5%) were females. At the end of the 2020–2021 season, the vaccination coverage rate against influenza among HCWs was 39.6% (2505) with a ∆% of plus 177.6 compared to the previous year. Among the 847 HCWs already immunized during the 2019–2020 season, 544 (64.2%) received the vaccination also during the 2020–2021 campaign. According to the multivariate logistic regression model, HCWs aged between 31 and 60 years had a higher likelihood of having received the immunization when compared with other age classes. Nurses (OR: 0.44–95% CI: 0.37–0.51), other HCWs (OR: 0.44–95% CI: 0.37–0.53) and administrative staff (OR:0.51–95% CI: 0.42–0.63) had a lower likelihood of being vaccinated when compared with physicians and residents. People already vaccinated in the previous flu season had a higher likelihood of being vaccinated compared with people not immunized in 2019–20 (OR: 3.17–95% CI: 2.71–3.71).

### 3.4. Responses to the Survey

A total of 673 HCWs out of 6323 (10.6%) filled out our survey. Characteristics of the respondents are shown in Table S1 reported in Supplementary Materials.

### 3.5. Flu Vaccination

Only 57.6% of the respondents considered themselves at higher risk of contracting influenza compared to the general population. Risk perception varied significantly among professional figures (*p* < 0.005), with the physicians, including residents, having the highest risk perception.

With regard to perceived importance of flu vaccination for the 2020/2021 season, 70.97% of the HCWs and 81.82% of resident doctors totally agreed that being vaccinated against influenza would be more important than the previous years because of COVID-19 emergency.

Almost half of the respondents (46.2%) stated that they had not received a flu jab during the previous vaccination campaign (2019/2020) due to a variety of different reasons (Table S2, in Supplementary Materials).

The measure most frequently suggested to improve vaccination uptake among healthcare professionals and residents was sending a personal invitation to all healthcare staff (37.5% of HCWs and 56.4% of residents), followed by raising the profile of the flu vaccination campaign among HCWs (35.2%) and improving the accessibility to the vaccination services among residents (43.6%). 

The overall vaccine attitude towards the flu vaccine was positive among respondents (median score = 22, range: 5–25). Evaluation of flu vaccine attitude among HCWs and resident doctors is presented in Figure S1 (in Supplementary Materials). 

In the multivariate analysis, “job category”, “risk perception” and “impact of pandemic on flu vaccine attitude” were significantly associated (*p* < 0005) with the attitude towards flu vaccination. Multivariate regression analysis results for vaccine attitude are shown in Table 4. In particular, being a physician or a resident, having a high-risk perception, having been vaccinated in the previous year and considering the flu vaccination more important than previous years due to COVID-19 emergency correlated with higher vaccination attitude scores.

### 3.6. COVID-19 Vaccination

Overall, vaccine attitude towards a novel COVID-19 vaccine was positive among respondents (median score = 19, range: 5–25).

Regarding a future COVID-19 vaccine, only 17.1% and 16.4% of HCWs totally agreed that the vaccine would be effective and safe. Figure S2 (in Supplementary Materials) shows the results obtained from the evaluation among respondents. The results of the multivariate analysis showed that having worked in a COVID-19 ward and being a physician, including being a resident, correlated with higher vaccine attitude scores.

Declared intention to receive the novel COVID-19 vaccine during the 2020/2021 season was high among respondents (70.8%).

## 4. Discussion

The aim of this study was to evaluate the impact of subsequent flu vaccination campaigns implemented in a large university hospital in Italy through the assessment of flu VC among HCWs and to investigate–by way of a survey–the perception toward the flu vaccine and a potential anti-COVID-19 vaccine in the same population and in the context of the COVID-19 pandemic. 

Starting from the 2018–2019 campaign, incremental initiatives to promote flu vaccination uptake were implemented. An increasing flu vaccination coverage rate among HCWs was registered. While contained in 2019–2020, the increase was significant in 2020–2021 as compared with the previous year (∆% = 177.6). The overall flu VC rate registered during the first two years of the study (11.6% and 14.3%) was slightly lower than the national average [8] (15.6%) in the same period. However, the overall flu VC rate rose to 39.6% in 2020/21, exceeding the median value of 30.2% reported by the ECDC in Europe during 2015–2017 vaccination campaigns [7].

Based on the multivariate logistic model, the likelihood of having received the immunization varied according to sex, age class, job category and having been vaccinated in the previous year, with some differences across the years. Data were partially confirmed by the results of the survey, as highlighted below.

Only for the 2018/19 flu campaign, males had a higher likelihood of being vaccinated when compared to females, as reported in other studies. However, no significant differences were registered during the following two seasons, in line with our survey results on vaccine attitude, but in contrast with literature evidence [21,22].

Among the identified job categories, physicians were the professionals most willing to get vaccinated, consistent with other studies conducted in Europe and Italy [21,22]. These findings were corroborated by the survey, with physicians showing higher attitude scores in comparison with nurses, other HCWs and administrative staff, consistent with the literature evidence [23].

Unexpectedly, a significant difference in VC rate was observed between residents and physicians, with residents presenting lower coverage rates in all but the 2019/20 flu campaign [23]. This might be due to perceived suboptimal access to vaccination among residents due to employment conditions, as our survey suggests, or to lower risk perception among younger age individuals. However, we could not find literature evidence investigating VC in these two groups.

Considering age groups, the situation changed greatly over the study period. People older than 60 years had a significantly higher likelihood of receiving the flu jab when compared with younger people during the 2018/19 flu campaign, probably due to higher individual risk perception and in line with previous literature [24]. In the following years, the VC rate among the youngest people (<30) increased greatly, reaching the highest coverage rate in 2019/20 (23.7%; +90%), possibly as a result of the incremental vaccination promotion initiatives and heightened knowledge of flu vaccination importance. In 2020/21, coverage rates grew more among people of the intermediate age classes (+ 293% among people between 41 and 50). This may be explained by the fact that, during last campaign, VC rates rose greatly in those population groups marked by a low VC in previous years (i.e., females, intermediate age groups).

Having been vaccinated in the previous year represented the most important variable to predict likelihood of accepting the flu jab, as confirmed by our survey. Similarly, having been vaccinated in the previous year correlated with a higher flu vaccine attitude score. However, while people already vaccinated in 2018/19 were 13 times more likely to get the flu jab during the 2019/20 campaign compared with people not immunized; people vaccinated in 2019/20 were only 3 times more likely to get the flu jab in 2020/21. This could be partially explained by the exceptional increase in the overall flu VC rate in 2020/21 compared to the previous year. Even considering the implementation of exceptional measures (e.g., increase in number of vaccination sites and dedicated staff), it is unlikely that the success of the 2020/2021 vaccination campaign could be solely attributed to optimized planning and communication. Most likely the COVID-19 pandemic played a crucial role for its success, as suggested by our survey results, with the majority of respondent HCWs believing flu vaccination to be particularly important in the 2020/21 flu season, due to the COVID-19 pandemic. In addition, the “impact of pandemic on flu vaccine attitude” variable significantly correlated with the flu vaccine attitude score, with people perceiving flu vaccination as more important due to the pandemic context showing a higher score. According to this data, the pandemic could have resulted in a heightened perception of the importance of flu vaccination among HCWs in the context of the pandemic, as other studies suggested [25,26], probably due to the risk of a possible co-circulation of the two viruses during flu season, as evidenced in the literature [27]. In support of this interpretation of the survey’s results, other studies state that the perceived level of a health threat is a strong predictor of people’s intention to adopt preventive behaviors, including undertaking flu vaccination [28,29].

Surprisingly, having worked in a COVID-19 ward during the pandemic was not associated with higher flu vaccine attitude scores. On the other hand, and in line with what previously described for vaccination coverages, having been previously vaccinated against influenza correlated with a higher flu vaccine attitude score. This result is consistent with what has been already reported by various authors in different contexts [30] and in a systematic review by Schmid et al. (2017) that highlighted the impact of previous decisions regarding flu vaccination on future choices [31].

As regards to a potential anti-COVID-19 vaccine, a poorer attitude was registered among HCWs-result in line with the literature evidence [32]-with only a few of them totally agreeing with the statements regarding importance to protect themselves (36.2%), safety (17.2%) and importance of being vaccinated to protect patients’ safety (38.3%). Other studies highlighted that the hesitancy towards COVID-19 vaccines is mostly driven by vaccine safety concerns [33].

According to the multivariate analysis, anti-COVID-19 vaccine attitudes varied significantly based on job category and having worked in a dedicated COVID-19 ward during the pandemic. With regard to the second variable, the fact that healthcare workers who had a more direct contact with COVID-19 patients had also a better attitude towards the vaccination against COVID-19 could be explained taking into account that the perception of health risks directly affects behaviors that help to prevent those risks [34]. HCWs perception towards anti-COVID-19 vaccines may have been changed after the beginning of COVID-19 vaccination program, as another study suggested for the community [35]. 

Our study had some limitations. Limited availability of flu vaccines during the 2020/21 flu campaign has threatened the success of the campaign itself, with interruption of vaccinations in mid-November due to vaccine shortage. As a consequence, flu vaccination coverage rates among HCWs of our hospital might have been higher if vaccines were always available. As regards to the survey, only 10.6% of AOUP’s HCWs responded to it. In addition, almost half of them had been vaccinated in the previous year suggesting a likely selection bias, with HCWs vaccinated with the flu jab in the previous season (2019/2020) being overrepresented in the sample. Furthermore, distribution of respondents by professional category did not reflect actual composition of AOUP staff, with nurses being the least represented group (only 6.5% responded to the survey).

## 5. Conclusions

In conclusion, a significant increase in flu VC among HCWs was observed in 2020/21, especially among those HCWs categories characterized by lower VC rates in the previous years. Despite the implementation of tailored interventions, the success of the 2020/21 flu campaign could be more likely attributed to the impact of COVID-19 pandemic on perception and attitude towards vaccination than to its good planning and communication. Our data showed that a concrete health threat, like a pandemic, could positively affect vaccine attitudes. These findings further underline the need for improving HCWs health and vaccine literacy and their ability to weighing personal and occupational risks and benefits in their choices regarding vaccination. Higher level of hesitancy towards a potential anti-COVID-19 vaccine was observed as compared to flu vaccine. However, these findings are time and context specific, and reflect the perception of a subgroup of HCWs in a time during which COVID-19 vaccines were still under development. 

## Figures and Tables

**Table 1:** Strategies implemented for the improvement of the Flu Vaccination Campaigns in the 2018/19, 2019/20 and 2020/21 flu seasons.

Strategy	Flu Vaccination Campaigns		
	2018/19	2019/20	2020/21
Informative material	Promotional material such as fliers, placed in strategic areas. Media coverage of the vaccination	Same as the previous season	Same as the previous season
Email invitations and reminders	Invitations and reminders sent to all employees	Same as the previous season	Same as the previous season
On-site vaccinations	6 ambulatories, divided equally in the two locations of Pisa’s hospital	Same as the previous season	Increase of the OSV
Occupational health vaccinations	2 ambulatories, divided equally in the two locations of Pisa’s hospital	Same as the previous season	Same as the previous season
Staff management (doctors, nurses)	8 nurses and 3 physicians	Same as the previous season	8 nurses and 8 physicians per day
Vaccination campaign timing	From October to February	Same as the previous season	Intensive vaccination campaign concentrated mostly in 15 days in October. On-demand availability until December
Opening hours	Monday to Friday morning for OHV. OSV rotating availability on Monday, Wednesday, and Friday afternoon	Same as the previous season	OHV and OSV opened all day from Monday to Friday for the 15 days of the intensive vaccination campaign

**Table 2:** Descriptive analysis results of vaccinated subgroups of the study population during the 2018/2019, 2019/2020 2020/2021 seasons and vaccine coverage rates.

Variables	2018/19				2019/20				2020/21				Δ%	
Age	Vaccinated	Total HCW	%	*p*-Value	Vaccinated	Total HCW	%	*p*-Value	Vaccinated	Total HCW	%	*p*-Value	Δ% from 2018/19 to 2019/20	Δ% from 2019/20 to 2020/21
<30	94	754	12.5	<0.0001	198	837	23.7	<0.0001	387	1116	34.7	0.0003	89.8	46.6
31–40	102	1090	9.4		158	1122	14.1		527	1294	40.7		50.5	189.2
41–50	131	1568	8.4		160	1631	9.8		635	1646	38.6		17.4	293.3
51–60	228	1739	13.1		238	1801	13.2		768	1787	43.0		0.8	225.2
>60	108	570	18.9		93	545	17.1		188	480	39.2		−9.9	129.5
**Sex**														
males	289	1753	16.5	<0.0001	339	1872	18.1	<0.0001	852	1990	42.8	0.0004	9.8	136.4
females	374	3968	9.4		508	4064	12.5		1653	4333	38.1		32.6	205.2
Job														
physicians	212	904	23.5	<0.0001	247	1062	23.3	<0.0001	638	1140	56.0	<0.0001	−0.8	140.6
nurses	146	2065	7.1		172	2030	8.5		719	2150	33.4		19.8	294.7
non-medical staff	19	157	12.1		20	163	12.3		90	159	56.6		1.4	361.3
administrators	85	623	13.6		78	620	12.6		236	607	38.9		−7.8	209.0
other healthcare workers	88	1123	7.8		102	1149	8.9		420	1233	34.1		13.3	283.7
residents	113	849	13.3		228	912	25.0		402	1034	38.9		87.8	55.5
Vaccinated in the previous year														
yes	np	np			362	632	57.3	<0.0001	544	833	65.3	<0.0001		14.0
no	np	np			485	5304	9.1		1961	5490	35.7			290.6
**Total**														
	663	5721	11.6		847	5936	14.3		2505	6323	39.6		23.1	177.6

**Table 3:** Predictor variables of having received the flu vaccine (yes/no) during the related flu influenza season according to the multivariate logistic model analysis.

Variables		2018/19			2019/20			2020/21		
		Odds Ratio	95% CI	*p* > z	Odds Ratio	95% CI	*p* > z	Odds Ratio	95% CI	*p* > z
**Sex**	Males	1			1			1		
	Females	0.67	(0.57, 0.80)	0.000						
**Age class**	<30	1			1			1		
	31–40	0.87	(0.61, 1.24)	0.436	0.67	(0.49, 0.91)	0.011	1.35	(1.11, 1.64)	0.002
	41–50	0.90	(0.60, 1.35)	0.614	0.53	(0.37, 0.76)	0.001	1.39	(1.13, 1.71)	0.002
	51–60	1.39	(0.94, 2.07)	0.097	0.60	(0.42, 0.86)	0.005	1.55	(1.26, 1.92)	0.000
	>60	2	(1.08, 2.55)	0.022	0.61	(0.40, 0.93)	0.021	1.03	(0.78, 1.35)	0.859
**Job category**	Physicians	1			1			1		
	Nurses	0.30	(0.24, 0.38)	0.000	0.40	(0.31, 0.51)	0.000	0.44	(0.37, 0.51)	0.000
	Non-medical staff	0.54	(0.32, 0.90)	0.018	0.47	(0.27, 0.80)	0.006	1.15	(0.82, 1.62)	0.418
	Administrators	0.53	(0.40, 0.70)	0.000	0.49	(0.36, 0.66)	0.000	0.51	(0.42, 0.63)	0.000
	Other HCWs	0.33	(0.25, 0.43)	0.000	0.41	(0.31, 0.54)	0.000	0.44	(0.37, 0.53)	0.000
	Residents	0.66	(0.45, 0.97)	0.031	0.95	(0.67, 1.35)	0.774	0.59	(0.47, 0.73)	0.000
**Vaccinated in the previous year**	No	_	_	_	1			1		
	Yes	_	_	_	12.84	(10.60, 15.56)	0.000	3	(2.71, 3.71)	0.000

**Table 4:** Predictor variables of the Flu vaccine attitude in HCWs according to the multivariate regression analysis.

Flu Vaccine Attitude						
Multivariate Regression Analysis						
Variables		*n*	%	Coeff.	95% CI	*p*-Value
Job category	Physicians	160	33.90	ref		
	Nurses	131	27.75	−0.90	(−1.53, −0.26)	0.006
	Other HCWs	84	17.80	−0.94	(−1.66, −0.22)	0.010
	Administrative staff	97	20.55	−1.39	(−2.09, −0.69)	0.000
Risk perception	Lower/Equal	219	46.40	ref		
	Higher	253	53.60	0.93	(0.43, 1.45)	0.000
Importance of flu vaccine during COVID-19 pandemic	1	5	1.06	ref		
	2	16	3.39	7.10	(4.43, 9.76)	0.000
	3	35	7.42	8.43	(5.93, 10.92)	0.000
	4	81	17.16	10.5	(8.15, 12.96)	0.000
	5	335	70.97	13.8	(11.41, 16.13)	0.000

Among respondents, 83.9% declared intention to receive the flu jab during the 2020/2021 season.

## Data Availability

Not applicable.

## Supplementary Materials

The following are available online at https://www.mdpi.com/article/10.3390/vaccines9070769/s1, Figure S1. Descriptive analysis of the Likert scale results concerning flu vaccine attitude: the scale range goes from 1—Totally disagree to 5—Totally agree, Figure S2. Descriptive analysis of the Likert scale results concerning COVID-19 vaccine attitude: the scale range goes from 1—Totally disagree to 5—Totally agree. Table S1: Characteristics of the HCWs respondents to the survey, Table S2: Main reasons reported by HCWs for not receiving flu vaccination in the previous year.
