# Supplementary material for: Evaluation of Flu Vaccination Coverage among Healthcare Workers during a 3 Years’ Study Period and Attitude towards Influenza and Potential COVID-19 Vaccination in the Context of the Pandemic
Source: Vaccines (Basel). 2021 Jul 9;9(7):769. doi: 10.3390/vaccines9070769 (PMC8310181; doi:10.3390/vaccines9070769)
Supplement: Supplementary file 1 [file vaccines-09-00769-s001.zip › vaccines-1277088-supplementary.pdf]

Evaluation of Flu Vaccination Coverage among Healthcare Workers during a 3 Years' Study Period and Attitude towards Influenza and Potential COVID-19 Vaccination in the Context of the Pandemic

Supplementary Materials

Table S1. Characteristics of the HCWs respondents to the survey

**Variables**	**HCWs**	
**Age**	**N**	**%**
30	118	18.5
31-40	145	22.8
41-50	135	21.2
51-60	177	27.8
60	62	9.7
**Sex**		
Males	203	31.9
Females	434	68.1
**Job Category**		
Physicians	160	25.1
Nurses	131	20.6
Other HCWs	84	13.2
Administratives	97	15.2
Residents	165	25.9
**Vaccinated in the previous year**		
Yes	311	48.8
No	326	51.2
**Ward**		
Administration	82	12.9
Diagnostic and Services	156	24.5
Surgical Ward	107	16.8
Medical Ward	292	45.8
**Work in a COVID ward**		
Yes	481	75.5
No	156	24.5
**TOTAL**	637	100.0

Table S2. Main reasons reported by HCWs for not receiving flu vaccination in the previous year

What are the main reasons why you have not been vaccinated?	N	%
It has never been proposed to me	43	13.8
It was proposed to me, but I was not given adequate information about it	8	2.6
It was proposed to me, but I forgot to undertake it	48	15.4
The proposed schedule and modalities were incompatible with my shifts	42	13.5
The risk linked to influenza does not justify the use of vaccination	44	14.1
Suboptimal efficacy of the vaccine	31	10.0
Fear of side effects	30	9.6
Fear of needles	7	2.3
I am not part of a risk category	45	14.5
I do not believe I pose a risk to my patients or colleagues	17	5.5
I never get sick with the flu	74	23.8
I am opposed to vaccines in general	7	2.3
TOTAL	311	127.3%*

**respondents could select more than one options, so the total percentage overcame 100.0%*

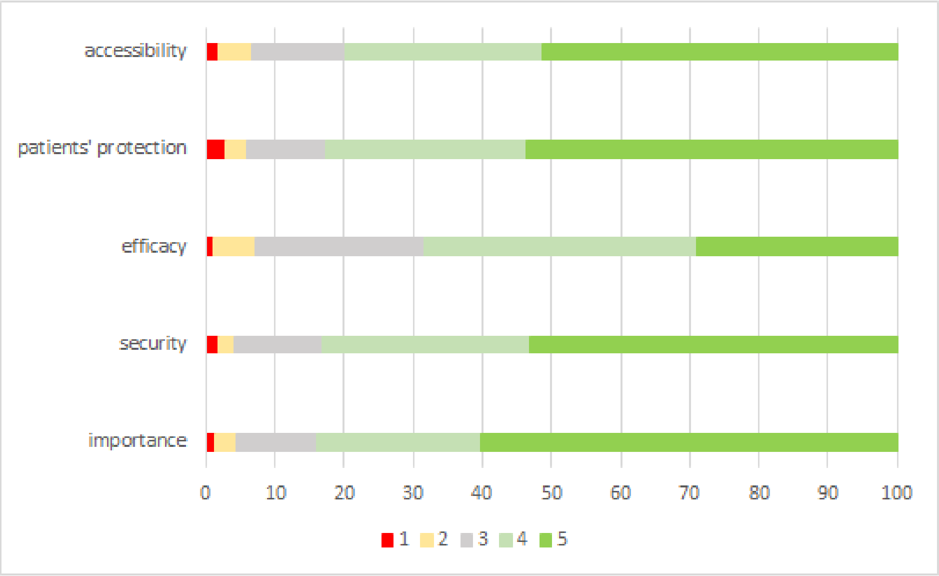

Figure S1. Descriptive analysis of the Likert scale results concerning flu vaccine attitude: the scale range goes from 1-Totally disagree to 5-Totally agree

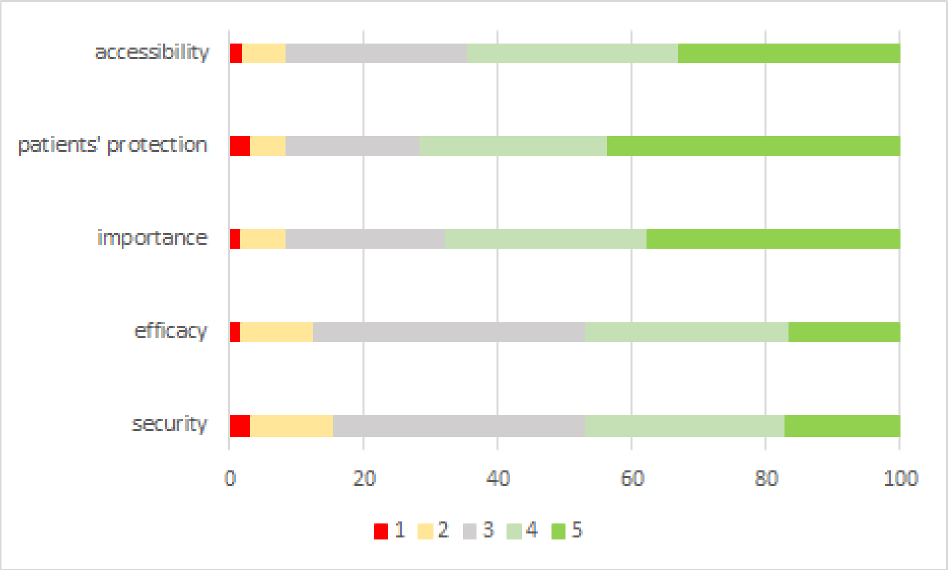

Figure S2. Descriptive analysis of the Likert scale results concerning COVID-19 vaccine attitude: the scale range goes from 1-Totally disagree to 5-Totally agree
